# Phase Evaluation, Mechanical Properties and Thermal Behavior of Hot-Pressed LC-YSZ Composites for TBC Applications

**DOI:** 10.3390/ma15082839

**Published:** 2022-04-12

**Authors:** Milan Parchovianský, Ivana Parchovianská, Ondrej Hanzel, Zuzana Netriová, Amirhossein Pakseresht

**Affiliations:** 1 Centre for Functional and Surface Functionalised Glass, Alexander Dubček University of Trenčín, Študentská 2, 911 50 Trenčín, Slovakia; ivana.parchovianska@tnuni.sk (I.P.); amir.pakseresht@tnuni.sk (A.P.); 2 Institute of Inorganic Chemistry, Slovak Academy of Sciences, Dúbravská Cesta 9, 845 36 Bratislava, Slovakia; ondrej.hanzel@savba.sk (O.H.); uachivaz@savba.sk (Z.N.)

**Keywords:** LC-YSZ composites, phase compositions, mechanical properties, thermal conductivity

## Abstract

In this work, La_2_Ce_2_O_7_-yttria-stabilized zirconia (LC-YSZ) composites with different weight fractions of YSZ (40–70 wt.%) were prepared by hot pressing at 1400 °C and investigated as a material for thermal barrier-coating (TBC) applications. For this purpose, the effect of YSZ addition on the phase composition, microstructure, mechanical performance and thermal behavior was studied. X-ray diffraction analysis showed that the LC-YSZ composites were mainly composed of a cubic ZrO_2_ and La_2_O_3_-CeO_2_-ZrO_2_ solid solution with a pyrochlore structure, indicating that the reaction between LC and YSZ took place during hot pressing. Scanning electron microscopy revealed the high microstructural stability of the prepared composites, as the pore formation was significantly controlled and a high relative density (>97%) was obtained. The microstructure of LC-YSZ bulk samples was relatively fine-grained, with an average grain size below or very close to 1 µm. YSZ doping improved the Vickers hardness of the LC-YSZ composites; the highest hardness, with value of 12 ± 0.62 GPa, was achieved for the composite containing 70 wt.% of YSZ. The fracture toughness of LC-YSZ composites was in the range from 2.13 to 2.5 MPa·m^1/2^. No statistically significant difference in heat capacity or thermal conductivity was found between the composites with different content of YSZ. The results showed that LC-YSZ composites have relatively low thermal conductivities from room temperature (1.5–1.8 W·m^−1^·K^−1^) up to 1000 °C (2.5–3.0 W·m^−1^·K^−1^). This indicates that the prepared LC-YSZ composite materials are promising candidates for TBC applications.

## 1. Introduction

Thermal barrier coatings (TBCs) are currently used in modern gas turbines and diesel engines to provide thermal insulation against hot gases to improve the performance and efficiency of these machines [1,2,3]. The use of TBCs is expected to increase their operating temperature or eliminate the need for cooling. For this reason, the development of new TBC materials with a long life, low thermal conductivity and high temperature resistance is a major challenge in the field of new-generation turbine engines [4]. TBCs typically consist of a high-strength, creep-resistant Ni-based superalloy as a substrate [5,6,7]; an oxidation-resistant bond coat; and an yttria-stabilized zirconia-based ceramic top coat (YSZ). When selecting a suitable material for TBCs, it is necessary for the material to meet several basic criteria, such as low thermal conductivity, a minimal coefficient of thermal expansion (CTE) difference between the coatings and the substrate, and good phase stability. Recently, YSZ has been the most widely used material for the top layer in TBCs due to its excellent properties, such as high CTE, low thermal conductivity and high thermal shock resistance [8]. However, the greatest disadvantages of YSZ are the limited sintering temperature (<1200 °C) and phase transformations of YSZ, leading to the formation of cracks and the consequent failure of the coatings [9]. To increase the efficiency of TBCs, new materials with even lower thermal conductivities and high phase stabilities, which could be used at high temperatures for a long time, are required. The goal of these new materials is to increase the efficiency of thermal conversion, reduce resource consumption and increase the life of TBC systems. The new potential TBC materials are mainly based on oxides and aluminates doped with ZrO_2_ or those with a fluorite and pyrochlore structure [10,11,12], such as La_2_Zr_2_O_7_ [13], Gd_2_Zr_2_O_7_ [14], Yb_2_Zr_2_O_7_ [15] and La_2_Ce_2_O_7_ [16,17]. Most attention has been given to fluorite-type La_2_Ce_2_O_7_ (LC), a solid solution of La_2_O_3_ in CeO_2_. At present, a new TBC coating based on LC with higher thermal stability [18], lower thermal conductivity and higher CTE [19] compared to YSZ has been developed.

Ceramic materials with low thermal conductivity are suitable for applications requiring thermal insulation. The main purpose of developing enhanced next-generation TBC coatings is to reduce thermal diffusion and thermal conductivity of ceramic materials [20]. LC has great potential as a TBC material, attracting attention mainly due to its low thermal conductivity and ability to withstand high temperatures (>1200 °C). In addition, LC has a higher corrosion resistance in the presence of CaO-MgO-Al_2_O_3_-SiO_2_ molten salts (CMAS) compared to YSZ-based TBC [21]. However, the CTE of LC decreases in the temperature range of 200–400 °C. Moreover, LC has poor mechanical properties, especially low fracture toughness, which leads to a short service life in the case of a single-layer LC TBC [22]. The fracture toughness of ceramic materials is a critical factor in their lifetime. In the case of TBCs subjected to thermomechanical loading, a high fracture toughness is also required. The fracture toughness of ceramic materials indicates their ability to resist crack propagation. The initiation of a fracture in brittle materials is related to the fact that ceramic materials contain technological defects in the form of various pores, cavities, cracks, etc., which arise during their production and processing. An improvement in the fracture toughness of LC can be achieved by adding a secondary phase in the form of various particles, for example YSZ. Wang et al. [23] reported that using 3YSZ increased the fracture toughness of La_2_Zr_2_O_7_ ceramics. The mentioned result was ascribed to the phase transformation that occurred in the secondary phase. Ma et al. [24] reported that the addition of YSZ in the Gd_2_Zr_2_O_7_-3YSZ composite increased the fracture toughness of the composite by increasing the YSZ content. The observed result was attributed to the high intrinsic toughness of YSZ at elevated temperatures. Moreover, two-layer LC/YSZ coatings [25] have shown a much longer service life compared to single-layer LC and YSZ coatings [26]. Only a few publications concerning the phase composition and mechanical properties of LC/YSZ composites are available [27,28]. However, no work has focused on the study of LC/YSZ composites prepared by the hot-pressing technique.

The present study focuses on the preparation and characterization of the hot-pressed LC-YSZ composites that may be used in the field of ceramic materials for high-temperature applications. Several studies revealed that chemical stability between LC and YSZ degrades at elevated temperatures, which may prevent their application as TBCs. Therefore, the chemical reactivity of LC and YSZ and sintering behavior of bulk samples was investigated at high temperature and pressure via hot-press experiments. Moreover, the influence of different weight fractions of YSZ on the microstructure and phase composition of the LC-YSZ composites was studied. The results presented in this paper also introduce new information on the thermal behavior and mechanical properties of the LC-YSZ composites as potential materials for TBCs.

## 2. Materials and Methods

### 2.1. Preparation of Powders and Composites

Commercial YSZ (8 mol.% yttria-stabilized zirconia, Inframat, Manchester, CT, USA) and LC powder synthesized through solid-state reaction were used as feedstocks for the preparation of LC-YSZ mixed powders and bulk samples. In the typical solid-state synthesis of LC powder, stoichiometric amounts of La_2_O_3_ and CeO_2_ (both 99.95% purity, Alchimica, Praha, Czech Republic) were ball-milled in isopropanol (Centralchem, Bratislava, Slovakia) for 24 h. The obtained LC powder mixture was then heat-treated at 1400 °C for 6 h. The conventional method of homogenizing powders was used to prepare the LC-YSZ powder mixtures containing different wt. fractions (40–70 wt.%) of YSZ. The calculated amounts of LC and YSZ powders were weighed and homogenized in isopropanol for 24 h in a polyethylene duster using ZrO_2_ milling balls to achieve uniform dispersion of LC and YSZ powders. Then, the suspensions were heated on a hot plate with constant stirring to remove the excess isopropanol. The product was dried in an oven at 100 °C for 24 h, crushed in an agate bowl to break up the agglomerates and finally sieved through a 100 µm mesh screen. The designation of the prepared LC-YSZ mixed powders and sintered bulk samples according to the relevant wt. fraction of YSZ and LC is shown in Table 1.

LC-YSZ bulk samples were obtained by sintering LC-YSZ powder mixtures via hot-pressing using a high-temperature laboratory hot press (Clasic 0220 ZL, Clasic, Řevnice, Czech Republic) with graphite heating elements. The LC-YSZ powders were placed into a 20 mm diameter graphite die and uniaxially pressed at 1400 °C in a vacuum atmosphere. The maximum applied pressure of 30 MPa and an isothermal dwell time of 1 h were used. A BN layer was applied as an interface between the sample and the graphite die for high-temperature protection.

### 2.2. Microstructure Analysis

The density of the LC-YSZ bulk samples was measured using Archimedes method by dual weighing in air and deionized water. Relative densities were expressed as the ratio of the bulk density of the prepared samples to the theoretical density. The microstructural examination of the prepared LC-YSZ composites after sintering was performed on polished and thermally etched cross sections via scanning electron microscopy (SEM, JEOL JSM 7600F, Tokyo, Japan). The samples were embedded into synthetic resin and polished on a universal polisher (Ecomet 300, Buehler, Leinfelden-Echterdingen, Germany). After polishing, the samples were removed from the synthetic resin and used for thermal etching. Thermal etching of the polished cross-section was carried out in a horizontal furnace (Clasic HT1600, Clasic, Řevnice, Czech Republic) at 1300 °C for 1 h in order to delineate the grain boundaries. The average grain size was determined by the linear intercept method (software Lince, TU Darmstadt, Darmstadt, Germany). At least 200 grains were measured to obtain a statistically robust set of data. X-ray powder diffraction analysis (XRD, Panalytical Empyrean DY1098, Panalytical, BV, Almelo, The Netherlands) with a Cu anode and an X-ray wavelength of λ = 1.5405 Å was used to determine the phase composition of the prepared powder mixtures and hot-pressed bulk samples. Individual XRD patterns were recorded in the 2-theta interval of 10–80°. Semi-quantitative study of the phase composition of the hot-pressed LC-YSZ composites was performed using Rietveld refinement of XRD data.

### 2.3. Mechanical Properties Measurements

Mechanical properties, i.e., Vickers hardness (HV) and indentation fracture toughness (K_IC_), were determined on embedded and polished samples using a Vickers indenter (Micro Hardness Tester, WIKI 200, AFFRI, Wood Dale, IL, USA). The measurements were carried out at an indentation load of 5 N (0.5 kgf) using an observation objective lens with magnification of 500× and a high-resolution CCD camera with autofocus and autoreading measurements. The indentation dwell time was 10 s. The HV value was obtained from the automeasurement of the indent size and hardness calculation. The K_IC_ values were calculated from the length of the radial cracks of the Vickers indentations according to the formula proposed by Shetty [29], Equation (1):

(1) $$K_{\mathrm{IC}}\left(\mathrm{Shetty}\right)=0.0889\sqrt{\frac{\mathrm{HV}\;\cdot 1000\;\cdot F}{4\cdot L}},$$

where HV is the Vickers hardness [GPa]; F is the indentation load [N]; and L = c − d, where c is the length of the crack and d is the length of the diagonal. The average values of HV and K_IC_ for each sample were obtained from 10 measurements.

### 2.4. Thermal Properties Measurements

The thermal diffusivity (α) was measured by laser flash method (Model LFA 1000; Linseis GmbH, Selb, Germany) on disk samples 20 mm in diameter and approximately 2 mm in thickness. Before measurements, a thin graphite layer was sprayed on both sides of the samples to prevent any laser-beam reflection and to reduce the sensitivity of the IR sensor on the back side of the sample. The measurements were carried out in vacuum at the temperatures ranging from 20 °C to 1000 °C (with a step of 100 °C). At each temperature, at least three measurements were performed, and the results were averaged. Thermal analysis was carried out in argon atmosphere in the temperature range of 20–1000 °C with a heating rate of 20 °C·min^−1^ using the device Netzsch STA 449 F3 Jupiter. Specific heat capacities (Cp) were measured using a DSC sample holder type S. Finally, the thermal conductivity (λ) of the LC-YSZ composites was calculated for each sample according to Equation (2):

(2) $$\lambda =\;\rho \cdot \alpha \cdot C_{p},$$

where ρ is the density [g/cm^3^], α is the thermal diffusivity [cm^2^/s], and C_p_ is the specific heat capacity [J/(kg·K)].

## 3. Results and Discussion

### 3.1. X-ray Diffraction Analysis (XRD)

**Figure 1:**
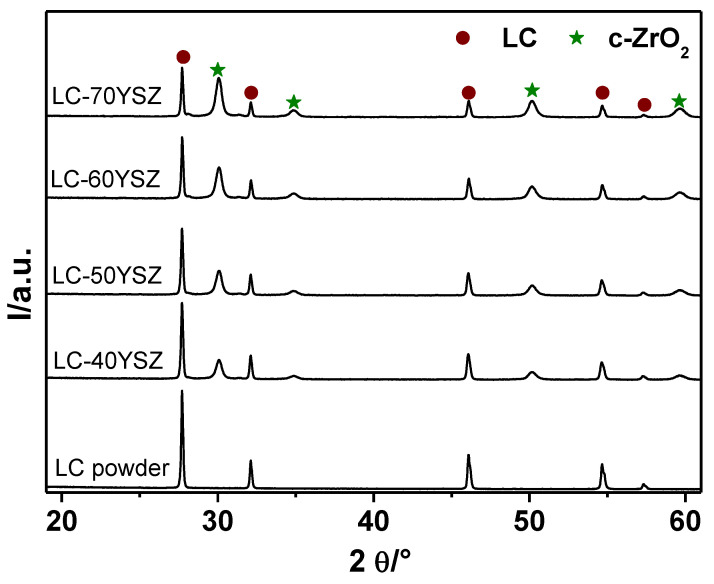
XRD patterns of LC-YSZ composite powders prepared by conventional mixing.

The crystal structure and phase evolution in both LC-YSZ mixed powders and sintered bulk samples were examined by XRD. Figure 1 shows the XRD patterns of the LC-YSZ composite powders prepared via conventional mixing. For comparison, the XRD pattern of a pure LC powder synthesized by solid-state reaction is also included in Figure 1.

LC is a solid solution of La_2_O_3_ in CeO_2_ with a fluorite-type structure [30]. The XRD pattern of LC is like that of undoped CeO_2_ with a small change of the lattice parameter [31]. This is due to the bigger ion radius of La^3+^ compared with that of Ce^4+^ [32]. The as-synthesized LC powder exhibits a defect fluorite structure since its XRD pattern matches well with the standard cubic phase of CeO_2_. This confirms that the calcination temperature of 1400 °C and heating time of 6 h were sufficient to form a single LC phase without the presence of unreacted La_2_O_3_. The fluorite structure of LC powder prepared by solid-state synthesis was also identified at 1300 °C and 1400 °C in the work of Dehkharghani et al. [33] and Zhang et al. [34], respectively. As can be seen in Figure 1, the diffraction patterns of all LC-YSZ mixed powders showed the presence of the fluorite-type LC phase and the YSZ powder, which is composed of cubic ZrO_2_ (c-ZrO_2_). No other impurity phases were detected after homogenization of the LC and YSZ powders. Figure 1 also shows that the intensities of the individual peaks of LC and c-ZrO_2_ phases are different depending on the quantity of primary powders used in the LC-YSZ composite powders. As expected, the intensities of the c-ZrO_2_ peaks for the individual compositions increased when increasing the wt. fraction of YSZ powder. In contrast, the intensities of the diffraction peaks belonging to LC gradually decreased as the amount of YSZ in the LC-YSZ mixed powders increased.

**Figure 2:**
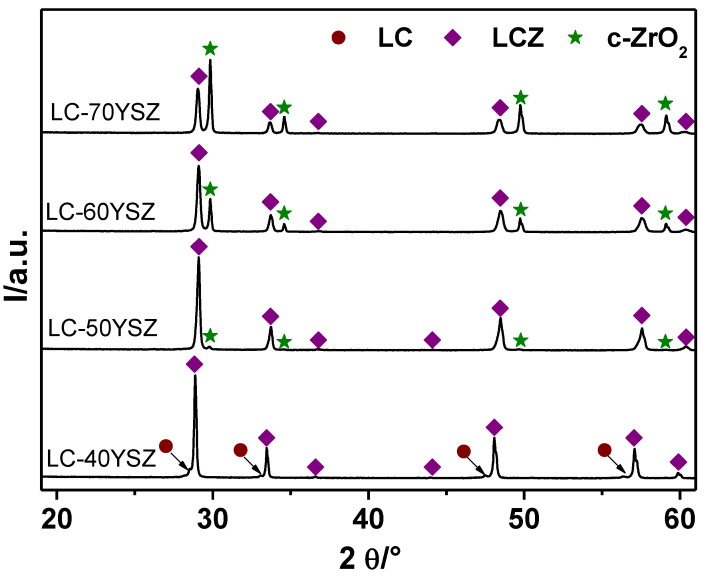
XRD patterns of the LC-YSZ bulk samples after hot pressing at 1400 °C.

Several authors investigated the chemical stability between LC and YSZ. Ma et al. [35] found that LC powders are chemically compatible with YSZ in the temperature interval of 200–1300 °C. Xu et al. [36] studied potential interactions between LC and YSZ in LC-YSZ composite materials after heat treatment at 1300 °C. It was reported [36] that a reaction between LC and YSZ occurred, with the reaction products being La_2_Zr_2_O_7_ (LZ) and CeO_2_. Zhang et al. [34] found that when the YSZ content in LC-YSZ composite coating is less than 40 mol.%, the newly emerging LZ integrates into the LC lattice, and the main phase is fluorite LC with a small amount of LZ in solid solution. However, when the YSZ content approaches 60 mol.%, pyrochlore LZ becomes the dominant phase with a low amount of LC in solid solution. The secondary phase is fluorite LC with a trace of LZ in solid solution [34]. To address this issue, the chemical reactivity between LC and YSZ in hot-pressed LC-YSZ bulk samples was investigated by XRD, as shown in Figure 2. Evidently, the reactions occurred between YSZ and LC during the hot pressing of LC-YSZ mixed powders, since a new pyrochlore phase, La_2_O_3_-CeO_2_-ZrO_2_ (LCZ), was observed in all XRD patterns (Figure 2). In the case of LC-40YSZ, the pyrochlore LCZ became the dominant phase, with typical low-intensity pyrochlore peaks located at 36.7° and 44.1° of 2θ angles. Besides the main LCZ phase, a certain amount of the initial fluorite LC solid solution was also observed in the LC-40YSZ pattern with the weak peaks located at the left side of the LCZ pyrochlore phase. This is due to the larger radius of Ce^4+^ (0.087 nm) compared to Zr^4+^ (0.072 nm) [34]. This indicates that the pyrochlore LCZ and fluorite LC structures coexist in the LC-40YSZ sample sintered at 1400 °C. Moreover, no characteristic peaks corresponding to c-ZrO_2_ were observed in LC-40YSZ pattern. This indicates that a solid-solution reaction between LC and YSZ occurred. As a result, all Zr^4+^ cations entered the LC lattice with a subsequent formation of LCZ solid solution. The XRD pattern of LC-50YSZ also showed the formation of an LCZ pyrochlore structure as the main phase, while the peaks corresponding to the LC fluorite phase completely disappeared. The absence of LC peaks in LC-50YSZ indicates that the LC solid solution was completely consumed throughout the reaction, resulting in the formation of a more stable substance, namely LCZ solid solution.

Importantly, small diffraction peaks corresponding to c-ZrO_2_ appeared in LC-50YSZ. When the YSZ content was 60 wt.%, the intensity of the LCZ phase decreased, while the intensity of the diffraction peaks corresponding to c-ZrO_2_ gradually increased. As the concentration of YSZ reached 70 wt.%, c-ZrO_2_ became the main phase while the secondary phase was LCZ solid solution. This is due to the fact that only a small amount of c-ZrO_2_ was involved in the reaction because of the lower content of LC powder (30 wt.%) in LC-70YSZ sample. As a result, a higher amount of c-ZrO_2_ remained unreacted compared to the other investigated LC-YSZ composites. In addition, all diffraction peaks corresponding to LCZ were shifted to higher diffraction angles compared with the XRD pattern of the LC solid solution. This can be explained by the Ce^4+^ replacement with the smaller Zr^4+^. The peaks corresponding to c-ZrO_2_ also showed slight shifting; however, the shift was to lower diffraction angles. Therefore, we assume that the small amount of La and/or Ce cations from the original LC/LCZ lattice interchanged with Zr^4+^ ions in YSZ lattice. As can be noticed in Figure 2, the characteristic pyrochlore peak at ~44.1° cannot be seen in LC-60YSZ and LC-70YSZ bulk samples because of the weaker intensities of LCZ pyrochlore peaks. Furthermore, the diffraction peaks of the c-ZrO_2_ in LC-60YSZ and LC-70YSZ became sharper compared to those of their powder-mixture counterparts, implying better crystallinity.

**Figure 3:**
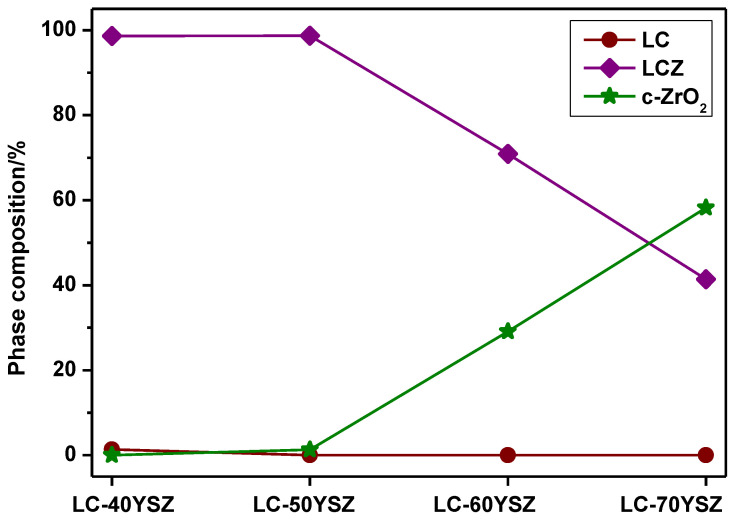
Phase composition of LC-YSZ composites after hot pressing, determined by the Rietveld refinement of XRD patterns.

The Rietveld analysis was utilized to calculate the quantity of newly generated phases during hot pressing (Figure 3). The results confirm (Figure 3) that the content of LCZ, which formed as a result of chemical reactions between LC and YSZ, decreased as the amount of YSZ in composites increased. In contrast, an increase in c-ZrO_2_ content was detected. The LC phase vanished in LC-YSZ composites containing more than 40 wt.% of YSZ, as demonstrated by the XRD patterns (Figure 2).

In zirconia-based materials, stabilizers such as CeO_2_ or Y_2_O_3_ can either precipitate from the material at a temperature below 1200 °C or selectively diffuse into c-ZrO_2_ at 1400 °C [37]. In this work, the effect of Y_2_O_3_ was not considered in the discussion due to its low concentration. The results discussed above agree with a study by Liu et al. [27]; the authors found that the interaction between YSZ and LC produces the LCZ solid solutions with pyrochlore or fluorite structures. Similar results were reported in the work of Zhang et al. [38], who studied the thermal stability and thermophysical properties of Ta_2_O_5_ doped La_2_Ce_2_O_7_ (LCT) spray powder and TBC coating. No characteristic peaks corresponding to Ta_2_O_5_ were observed in LCT diffraction patterns, implying that Ta^5+^ entered the LC lattice, resulting in the formation of LCT solid solution.

### 3.2. Relative Density and Microstructure Characterization

Bulk density is one of the most important parameters of composite ceramic materials for various construction applications. The usual requirement is that the bulk density of the material is identical or close to the theoretical density of the composite ceramic material, i.e., the composite material should have virtually zero porosity. As can be seen in Table 2, the relative density of all hot-pressed LC-YSZ bulk samples was relatively high, indicating that the addition of YSZ particles has a significant effect on the compaction. The applied sintering conditions were sufficient to achieve a relative density close to the theoretical density of the prepared composites. The highest relative density of 98.6% was found for the LC-70YSZ bulk sample. As for the LC-YSZ bulk samples containing 40–60 wt.% of YSZ, relative densities of approximately 97% were obtained. Generally, YSZ and LC have poor sintering resistance, which is associated with the rapid shrinkage of materials and with the closing of pores [16]. Cao et al. [16] also reported that LC-based materials have low sintering resistance at temperatures above 1280 °C. However, Liu et al. [27] reported that both the amount and size of pores in LC-YSZ composites increased significantly with increasing sintering temperature, indicating the improved sintering resistance of the samples.

**Figure 4:**
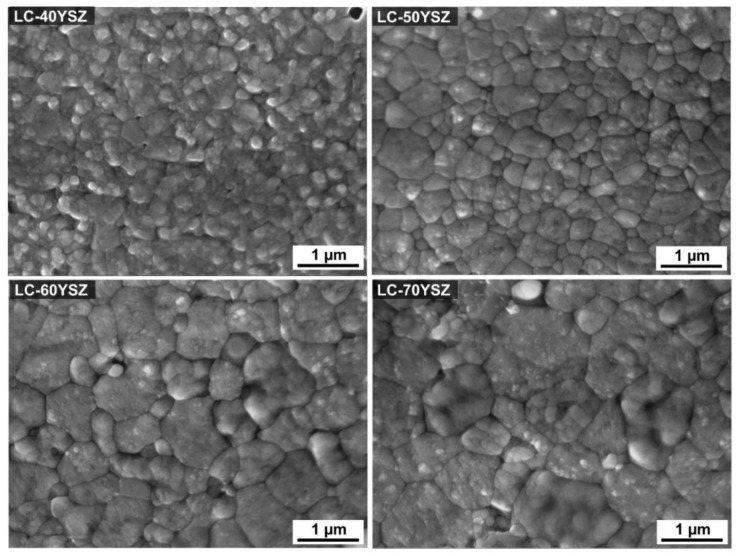
The SEM microstructures of hot-pressed LC-YSZ composites.

**Figure 5:**
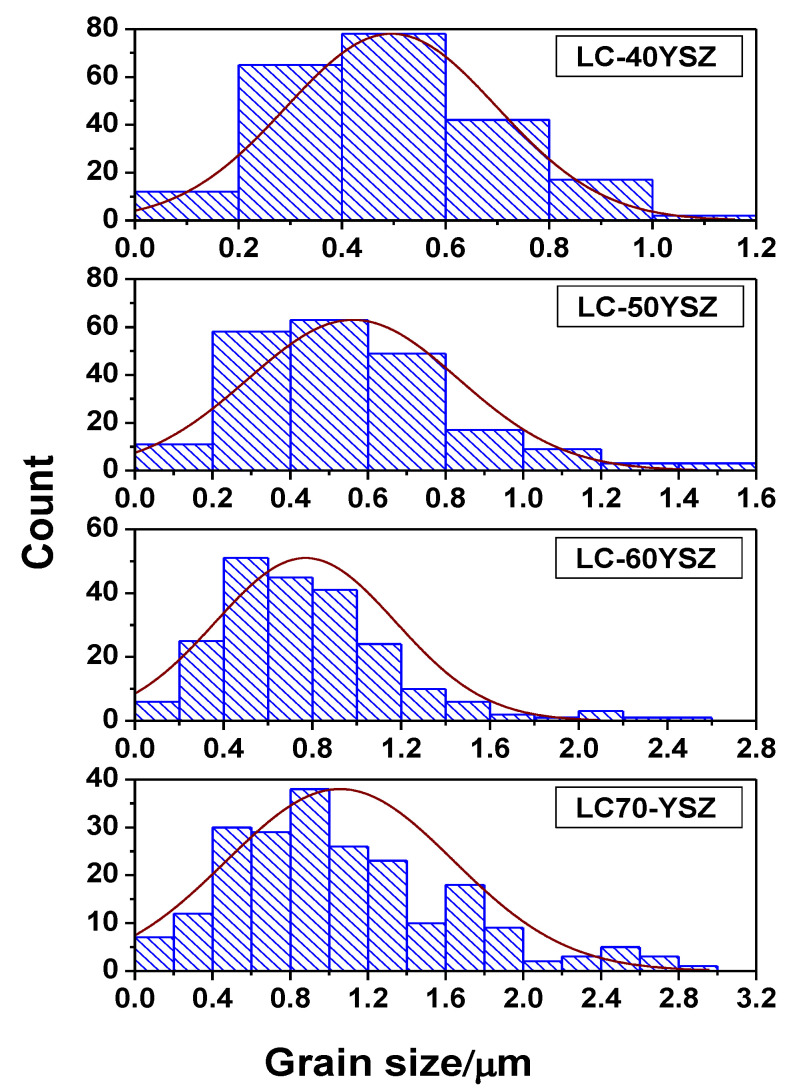
Grain-size distribution of hot-pressed LC-YSZ composites.

The microstructures of thermally etched LC-YSZ composite samples hot-pressed at 1400 °C are presented in Figure 4. The purpose of thermal etching was to reveal grain boundaries and to determine the average grain size. Grain-size distribution of hot-pressed LC-YSZ composites is shown in Figure 5. As can be seen in Figure 4, interfaces and boundaries between the individual grains can be clearly observed in the hot-pressed LC-YSZ samples. All LC-YSZ bulk samples are composed of fine grains that are considered to mainly be YSZ and LCZ, as shown by XRD analysis. As illustrated in Figure 4, grain coarsening and grain growth can be observed with the increasing wt. fraction of YSZ in the LC-YSZ bulk samples. As shown in Table 2 and Figure 5, the average grain size in LC-40YSZ sample is 0.5 ± 0.2 µm, while the average grain size increased to 1.1 ± 0.6 µm in LC-70YSZ. Furthermore, both large and small grains can be observed in LC-60YSZ and LC-70YSZ samples, which might be associated with the higher content of YSZ and subsequent consumption of small LCZ grains during sintering. A small quantity of pores can be observed in some samples, which is in accordance with the results of the relative densities of the bulk samples shown in Table 2.

The random occurrence of pores can be explained by the presence of agglomerates that may have formed during the homogenization and drying of mixed powders. Agglomerates that are not removed before sintering are then present in the form of nonsinterable inclusions that block movement at grain boundaries and prevent compaction. We assume that these agglomerates might suppress compaction, resulting in pore formation in the sintered materials. Notably, pores in the LC-70YSZ composite were rarely observed, indicating a nearly full densification of the sample. Xu et al. [36] examined the formation of pores in plasma-sprayed LC/YSZ composite coatings and ascribed these pores to the Kirkendall effect [39,40]. In the study by Liu et al. [27], the pore formation in LC/YSZ composite samples after high-temperature treatment was also ascribed to the Kirkendall effect. Furthermore, the flux of vacancies from YSZ to LC, which compensate the differences in the diffusion rates of Ce^4+^ and Zr^4+^ cations, also contributed to the pores’ formation. Generally, the basic microstructure of ceramic composites consists of a matrix in which the secondary particles are scattered and located at the grain boundaries or inside the matrix. Grain growth in composite materials with good chemical compatibility is usually suppressed by the addition of a secondary phase. The secondary phase can be used to control abnormal grain growth, and this can be introduced by mixing a two-phase powder. This fact is due to the significant “pinning” effect of the secondary phase. This means that the average grain size of the matrix decreases with an increasing volume fraction of the secondary phase [41]. However, Liu et al. [27] reported that the reaction between YSZ and LC led to abnormal grain growth near their interface in the LC and LC/YSZ composites. For that reason, the authors analyzed the interdiffusion reaction between LC and YSZ according to the experimental results. It was reported [27] that during high-temperature treatment, elementary interdiffusion occurred at the LC/YSZ interface. As a result, Ce^4+^ (La^3+^) ions in LC had a tendency to dissolve into the YSZ lattice, and Zr^4+^ (Y^3+^) ions tended to dissolve into the LC lattice. The strong reaction between YSZ and LC was ascribed to the difference in Gibbs free energy. This resulted in a reorganization of pores and the generation of a reaction layer comprised of ZrO_2_-CeO_2_-La_2_O_3_. In the present study, we assume that the grain growth in LC-YSZ samples was also promoted by the reaction effect and interdiffusion between LC and YSZ particles.

### 3.3. Mechanical Properties

**Figure 6:**
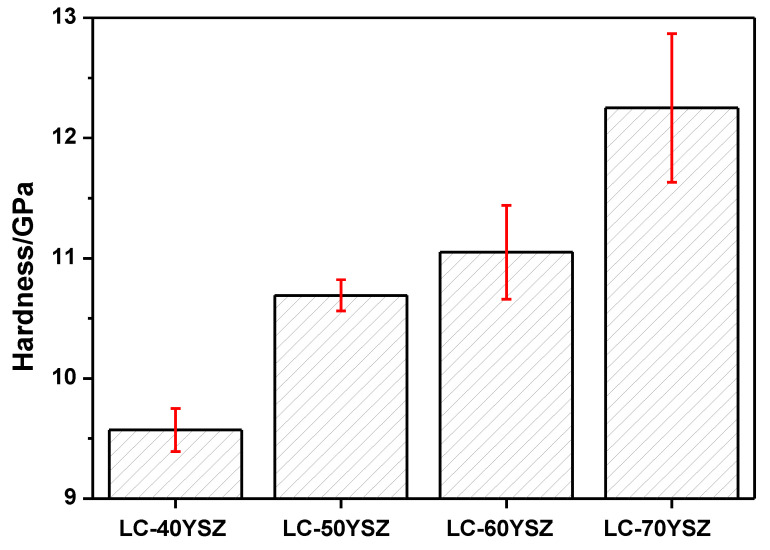
Vickers hardness of LC-YSZ composites.

Mechanical properties are an important factor influencing the durability of TBCs. High hardness and fracture toughness are especially necessary for the material to be used as a TBC because they can improve the erosion resistance and extend the lifetime. The Vickers hardness values of hot-pressed LC-YSZ composites are summarized in Figure 6. The evolution of their mechanical properties was evaluated mainly based on the influence of the wt. fraction of YSZ particles and the resulting microstructure of LC-YSZ composites. As obvious from Figure 6, there is a relationship between the wt. fraction of YSZ powder in the composite material and the Vickers hardness. Experimental results showed that the Vickers hardness of LC-YSZ composites increased gradually with an increasing content of YSZ particles. The LC-70YSZ composite material reached the highest hardness value (12 ± 0.62 GPa), while the hardness value of the LC-40YSZ bulk sample was 9.57 ± 0.18 GPa. The improvement of HV in the LC-60YSZ and LC-70YSZ samples can be attributed to the existence of a secondary YSZ phase, which generally has higher hardness than single-phase LC. Another important factor that could have a beneficial impact on the hardness of LC-YSZ composite materials is their relatively low porosity and fine-grained microstructure, with grain sizes in the submicron region. Generally, the hardness is inversely proportional to the porosity degree in composites. As already mentioned, the prepared LC-YSZ composites reached >97% of their relative density and only a few pores were observed from SEM images (Figure 4). Therefore, we assume that the residual porosity had a negligible effect on the hardness of the investigated composites. The standard deviations of HV for LC-40YSZ and LC-50YSZ samples, which range between 0.18 and 0.13, imply a homogeneous microstructure and uniform properties over the entire volume of the prepared composites. In contrast, the different mechanical properties of the LCZ and YSZ grains in LC-60YSZ and LC-70YSZ samples resulted in larger values of standard deviation (0.39 and 0.62).

**Figure 7:**
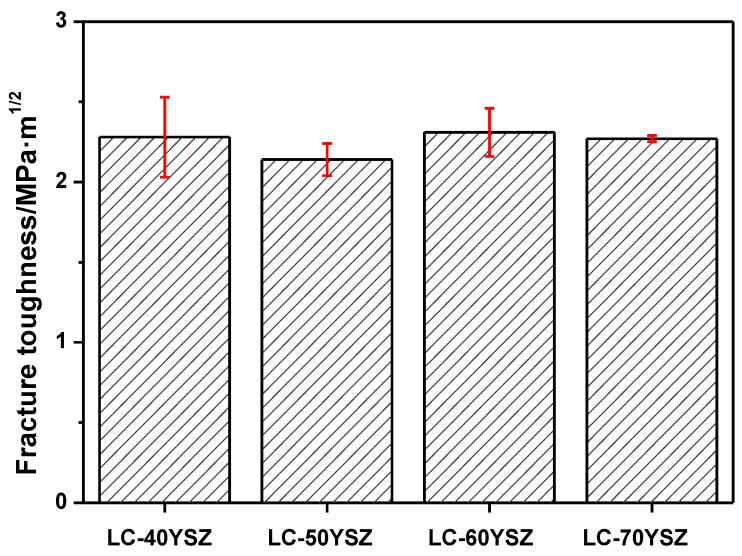
The fracture toughness of LC-YSZ composites.

The fracture toughness of LC-YSZ composites is shown in Figure 7. Regarding the determination of fracture toughness from indentation data, it is necessary to emphasize that the measured values are not absolute and can only serve, at best, to compare qualitatively similar materials. It can be seen from Figure 7 that the fracture toughness of LC-YSZ composites is in the range of 2.13 to 2.5 MPa·m^1/2^. Dependence of the fracture toughness on the wt. fractions of the YSZ particles do not show any clear trend. The lowest fracture toughness (2.14 ± 0.1 MPa·m^1/2^) was observed for the composite containing 50 wt.% of YSZ. The highest value of fracture toughness (2.31 ± 0.15 MPa·m^1/2^) was found for the composite containing 60 wt.% of YSZ. The measurements did not show any relationship between the fracture toughness and the LC grain size, which would indicate the presence of compaction mechanisms in the material.

It has been reported by other authors that YSZ has a positive influence on the fracture toughness of LC [33,34]. However, the mechanism by which the presence of YSZ particles should increase the fracture toughness of LC-YSZ composites is still not clear. Several toughening mechanisms could be responsible for the improvement of the fracture toughness in the LC-YSZ composites. These are ferroelastic toughening [42,43], t − m phase transformation toughening [23,34], an increased fracture energy arising from the chemical reaction between LC and YSZ [34,44] and a mismatch of CTE phases [23]. Mercer et al. [42] found that this is related to the metastable tetragonal phase (t′-ZrO_2_), which has been found to be highly stable up to 1400 K [43]. The nondiffusion shear transformation directly from the cubic phase (c-ZrO_2_) results in a ferroelastic t′-ZrO_2_ phase. This transformation could solidify the ceramics by reorienting the domain and does not transform directly into the monoclinic phase (m-ZrO_2_) during cooling [23,34]. According to Zhang et al. [34], the improved fracture toughness is mostly caused by the solid-solution reaction between YSZ and LC. The incorporation of YSZ into the LC lattice is thought to improve the lattice’s cohesive energy, which is proportional to the fracture energy [34]. High fracture energy, as it is well known, can lead to high fracture toughness [44]. The different CTEs of LC and YSZ can also contribute to the improved fracture toughness of LC/YSZ materials [23]. Residual stresses are generated in the ceramic matrix during the cooling process from the sintering temperature as a consequence of the difference of the CTEs of the two phases.

### 3.4. Thermal Properties

**Figure 8:**
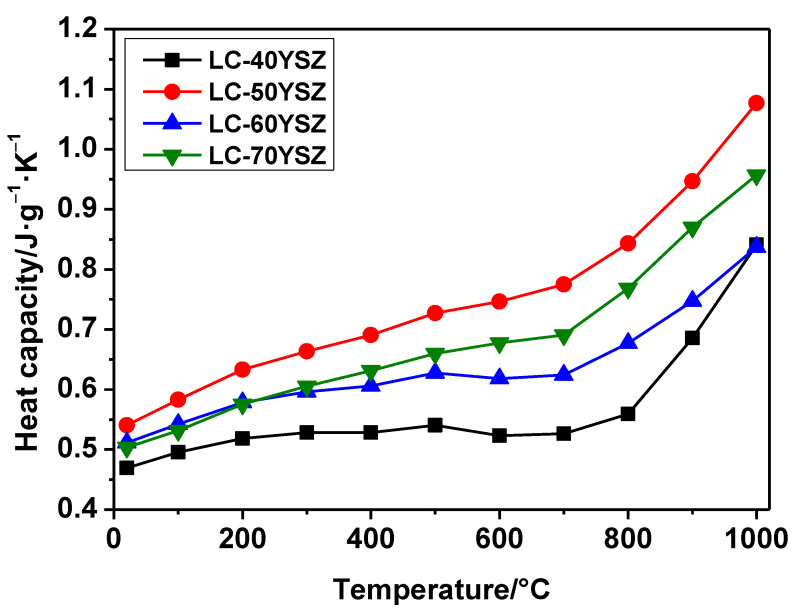
Temperature dependence of the specific heat capacities of the LC-YSZ composites.

Figure 8 shows the temperature dependency of the measured specific heat capacities of LC-YSZ composites. The specific heat capacities of the investigated LC-YSZ composites are in the range of 0.45–0.55 J/g·K and 0.8–1.1 J/g·K at temperatures of 20 and 1000 °C, respectively. As illustrated in Figure 8, all LC-YSZ composites show a similar behavior: the specific heat capacities increase as the temperature rises. Furthermore, the specific heat capacities of all compositions increase slightly from 20 °C up to 700 °C, followed by a rapid increase in specific heat capacities from 700 to 1000 °C. The specific-heat-capacity increase can be attributed to the volume increase and phonon excitation as the temperature increases [45]. Clearly, LC-50YSZ shows a higher specific heat capacity compared to other LC-YSZ composites. On the contrary, the lowest specific heat capacities were measured in the LC-40YSZ composite.

**Figure 9:**
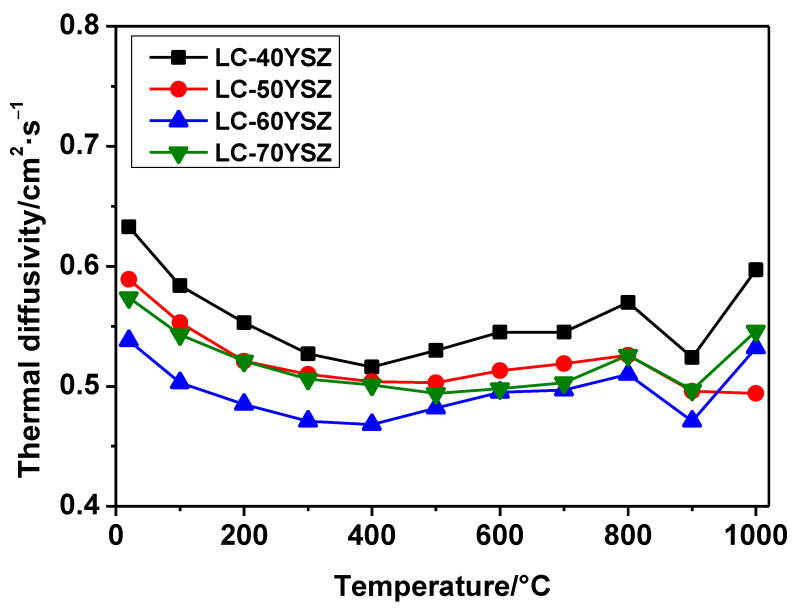
Temperature dependence of the thermal diffusivities of the LC-YSZ composites.

Figure 9 shows the temperature dependence of the thermal diffusivity of the LC-YSZ bulk samples measured in the temperature range of 20–1000 °C. As illustrated in Figure 9, the change in thermal diffusivity as the temperature increases is similar for all measured LC-YSZ samples. The thermal diffusivities of LC-YSZ composites at room temperature were in the range of 0.54–0.64 cm^2^·s^−1^. Subsequently, the thermal diffusivities decreased with temperature up to 400 °C with values in the range of 0.47–0.52 cm^2^·s^−1^, and then slightly increased up to 800 °C, achieving values in the range of 0.52–0.57 cm^2^·s^−1^. Furthermore, a sudden decrease in the thermal diffusivities was observed at 900 °C, while at 1000 °C the thermal diffusivities increased again, except for the LC-50YSZ bulk. The measured values of thermal diffusivities at 1000 °C were in the range of 0.5–0.6 cm^2^·s^−1^. The observed behavior can be attributed to the combination of lattice thermal conduction, radiative transport and/or disintegration of the graphite film.

**Figure 10:**
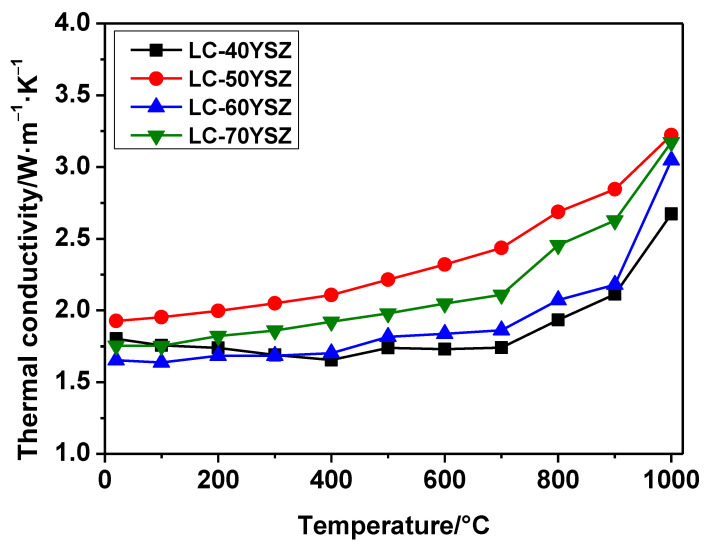
Temperature dependence of the thermal conductivity of the LC-YSZ composites.

The situation was different for the thermal conductivity, which was calculated for each composition from the measured values of the thermal diffusivity, specific heat, and bulk density according to Equation (2). The calculated thermal conductivities for the LC-YSZ composites as a function of temperature are shown in Figure 10. A similar trend of thermal conductivities to those of the specific heat capacities was observed for all LC-YSZ composites except for the LC-40YSZ sample. The thermal conductivities of the LC-50YSZ, LC-60YSZ and LC-70YSZ bulks gradually increased with increasing temperatures from 20 °C to 1000 °C. However, the thermal conductivity of LC-40YSZ slowly decreased with temperature up to 400 °C, and then increased as the temperature rose to 1000 °C. This is a rather unusual trend and is not consistent with the observations of thermal conductivity found by other authors [46,47,48] who report that the thermal conductivity of ceramic materials decreases with elevating temperature. LC-YSZ composites studied in this work are insulators, and thus the heat conduction is predominately by lattice vibrations or radiation. Since LC-YSZ composites are a heterogeneous material which may contain, e.g., residual porosity, the mentioned mechanism also contributes to the heat transfer. The most important factor influencing the thermal conductivity is phonon scattering. We assume that the presence of LC in LC-YSZ composites causes more serious lattice distortion, which provides more opportunities for phonon scattering. The phonons are also known to interact with lattice defects, grain boundaries, and other microstructure defects. Based on the XRD analysis, the crystal structure of the LC is a cubic defect fluorite structure. Many point defects introduced by La_2_Ce_2_O_7_ lead to the relatively strong phonon–phonon scattering resulting in the reduced thermal conductivity in LC-YSZ composites.

However, in this work the thermal conductivity increases with temperature. A similar trend in the thermal conductivity depending on the temperature of the composite ceramics was observed in the work of Zhang et al. [49] who investigated the thermal properties of three ceramic materials (La_2_Zr_2_O_7_/YSZ, YSZ, La_2_Zr_2_O_7_). In the work of Zhang et al. [49], an increase in the thermal conductivity of the investigated materials with increasing temperature was observed, while the thermal conductivity of the La_2_Zr_2_O_7_/YSZ composite ceramics was in the range of 1.77–2.30 W·m^−1^·K^−1^ (200–1000 °C). The authors [49] assumed that the different heat-diffusivity mechanisms created this result. A straight spreading process dominates the heat diffusivity at low temperatures, with spreading occurring in low (LC) and high (YSZ) thermal conductivity materials. A curved spreading mechanism, on the other hand, performs the thermal diffusivity work at the high temperatures, and heat spreads quickly in the high-conductivity material [49]. Therefore, YSZ influences the thermal conductivity of LC/YSZ composites at high temperature. In this work, the lowest thermal conductivity at room temperature was reached for the LC-60YZS sample (1.5 W·m^−1^·K^−1^). The highest thermal conductivity was recorded for the LC-50YSZ sample (1.8 W·m^−1^·K^−1^). The calculated values of thermal conductivity at 1000 °C range from 2.5 to 3.0 W·m^−1^·K^−1^, which are slightly higher than the values of thermal conductivities calculated at room temperature. The thermal conductivities measured at room temperature are comparable to the values for TBC materials reported by other authors [46,47,48], where the thermal conductivities were in the range of 1.5–2.2 W·m^−1^·K^−1^ for LZO [46,47], LCO [48] and YSZ [18], respectively. Therefore, it can be concluded that the thermal conductivities of the investigated LC-YSZ composites are relatively low, and therefore can be utilized as a material for TBCs.

## 4. Conclusions

The results presented in this paper provide a systematic study on the microstructure, phase composition and thermal and mechanical properties of LC-YSZ composites as a material designed for TBCs applications. XRD of LC-YSZ mixed powders before sintering showed the presence of two phases: c-ZrO_2_ and LC solid solution with a fluorite-type structure. Reactions occurred between the YSZ and LC during hot pressing of the LC-YSZ mixed powders, indicated by a new pyrochlore phase of La_2_O_3_-CeO_2_-ZrO_2_ (LCZ) observed in the XRD patterns. The prepared composites exhibit high relative density, and the microstructure of LC-YSZ composites was relatively fine-grained with an average grain size below 1.1 µm. The Vickers hardness of LC-YSZ composites increased with increasing mass fractions of YSZ particles. In contrast, the fracture toughness was not affected by the wt. fraction of YSZ and ranged between 2.13 and 2.5 MPa·m^1/2^. The thermal conductivity increased with increasing temperature, while the lowest thermal conductivity in the temperature range of 400–1000 °C was found for the LC-40YSZ bulk sample. The results of this study indicate the high potential of the investigated LC-YSZ composites for applications in the field of ceramic materials used for high-temperature barriers.

## Figures and Tables

**Table 1:** Designation of the prepared LC-YSZ compositions.

Composition	LC (wt.%)	YSZ (wt.%)	LC (mol.%)	YSZ (mol.%)
LC-40YSZ	60	40	44	56
LC-50YSZ	50	50	34	66
LC-60YSZ	40	60	26	74
LC-70YSZ	30	70	18	82

**Table 2:** Relative densities and average grain sizes of the investigated LC-YSZ bulk samples sintered at 1400 °C.

Sample	Relative Density (%)	Average Grain Size (µm)
LC-40YSZ	97.7	0.5 ± 0.2
LC-50YSZ	97.8	0.6 ± 0.3
LC-60YSZ	97.2	0.8 ± 0.4
LC-70YSZ	98.6	1.1 ± 0.6

## Data Availability

Not applicable.
